# Magnitude and determinants of gender-based violence among female students in Ethiopian higher educational institutions: a systematic review and meta-analysis

**DOI:** 10.3389/fpsyt.2024.1387032

**Published:** 2024-08-22

**Authors:** Gebresilassie Tadesse, Techilo Tinsae, Girum Nakie, Gidey Rtbey, Fantahun Andualem, Mulualem Kelebie, Getasew Kibralew, Asnake Tadesse Abate, Shegaye Shumet, Mamaru Melkam, Setegn Fentahun

**Affiliations:** ^1^ Department of Psychiatry, School of Medicine, College of Medicine and Health Sciences, University of Gondar, Gondar, Ethiopia; ^2^ Department of Neonatal Health Nursing, School of Nursing, College of Medicine and Health Sciences, University of Gondar, Gondar, Ethiopia

**Keywords:** gender-based violence, students, systematic review, meta-analysis, Ethiopia

## Abstract

**Background:**

Many adolescents are vulnerable to gender-based violence, and it is a major public health issue. Even though the burden of gender-based violence is still high in Ethiopia, there is a lack of summary information to address the problem. Therefore, this study aimed to assess the pooled magnitude and factors associated with gender-based violence among female students in Ethiopian higher educational institutions.

**Methods:**

The primary articles were searched using databases like PubMed, Google Scholar, CINAHL, SCOPUS, EMBASE, and African Journal Online. Articles that assessed the magnitude and factors associated with GBV among female students in Ethiopia were included. A Microsoft Excel spreadsheet was used to extract the data, which was then exported to Stata version 14 for further analysis. The statistical heterogeneity was evaluated using the I^2^ test. Due to heterogeneity, a random effect meta-analysis model was employed. Publication bias was checked through Egger’s weighted regression test and funnel plot.

**Results:**

This study included twenty-five primary studies with 13,013 participants. The prevalence of lifetime GBV (n = 7), sexual violence (n = 25), and physical violence (n = 7) was found to be 51.42% (42.38, 60.46), 46.53% (39.86, 53.21), and 37.93% (24.68, 51.18), respectively. Witnessing their mother’s abuse by their father during childhood, a lack of open discussion in the family about reproductive health and related personal issues, alcohol consumption, and tight family control were some of the factors significantly associated with lifetime gender-based violence. Furthermore, those who had drunken friends, a regular boyfriend, multiple sexual partners, and chat chewing were factors significantly associated with lifetime sexual violence.

**Conclusions and recommendations:**

Our findings revealed that half of female students at higher institutions suffered from violence. So it is recommended to provide accessible information about the consequences of GBV and early intervention for students with the above factors.

**Systematic review registration:**

https://www.crd.york.ac.uk/prospero/#recordDetails, identifier CRD42023494760.

## Introduction

Gender-based violence (GBV) is any detrimental conduct carried out against an individual’s will that is motivated by socially constructed distinctions between males and females. It is the most common kind of violence against women and girls in all dimensions, at all times, and at all levels, and against people of all ages, religions, and ethnicities (1). Any act of gender-based violence (GBV) that causes, or is likely to cause, physical, sexual, or psychological harm or suffering to women is considered violence against women (VAW). This includes threats of such acts, coercion, or arbitrary deprivation of liberty, whether the incident takes place in public or in private. GBV and VAW are sometimes used interchangeably since women are disproportionately affected (95% vs. 5%) in comparison to men (2–4). Violence against women is one of the most common human rights violations, which also interferes with national development and gender equality. GBV affects both sexes equally, yet it affects women more frequently than men (5).

Gender-based violence (GBV) can happen at home, on the streets, in prisons, workplaces, colleges, and other settings. Physical harm, long-term infections like HIV/AIDS, several negative reproductive and sexual health outcomes like unintended pregnancy, unsafe abortion, and vaginal discharge, as well as emotional and psychological stress that may lead to suicidal thoughts and acts, are all possible effects of GBV (6, 7). The World Health Organization reported that the lifetime and current (past 12 months) prevalence of physical or sexual intimate partner violence ranged from 15 to 71% and 4 to 54%, respectively, while the prevalence of emotional violence ranged from 20 to 75%, according to a multi-country study on violence against women (8). This finding indicated that the lowest prevalence has been reported in Japan, whereas the highest prevalence was reported in Ethiopia, Peru, and Bangladesh. Since the age of 15, between 0.3% and 12% of women have been coerced by non-partners to have sex or carry out sexual behavior they do not want (8). Victims of violence against women typically fail to report abuse due to their feelings of guilt, shame, and embarrassment (9). Globally, 1 in 5 women will be the victim of rape or attempted rape, and 1 in 3 women will experience sexual, physical, or psychological violence at some point in their lives (10). Even though one in five female college students has experienced attempted or completed rape at some point in their academic careers, less than 5% of them come forward to disclose having been raped (11). According to a systematic review and meta-analysis that identified 104 articles carried out in sixteen (12) different countries, the pooled prevalence of sexual violence among college students was 17.4% (13). Sub-Saharan Africa (SSA) has provided evidence of a high incidence of gender-based violence in educational institutions (14). According to data from the Global Based School Survey (GBSS), the extent of current physical and sexual violence in five African countries ranged from 27–50% and 9–33%, respectively (15, 16). African females encounter gender-based violence in a variety of contexts, such as the workplace and educational institutions (12). For example, a study conducted among female university students in Northern Nigeria found that the overall prevalence of gender-based violence was 58.8%, with physical, sexual, and emotional violence accounting for 22.8%, 22.2%, and 50.8% of cases, respectively (17). The overall pooled prevalence of lifetime gender-based violence among female youths in educational institutions in sub-Sahara was 52.83%, whereas the pooled estimate of sexual violence, physical violence, and emotional violence was 26.22%, 18.86%, and 27.06%, respectively (14). Studies carried out in three South African universities (12), a Southeast Nigerian university (18), and a Ugandan university (19) showed that the burden of GBV was 57.8%, 34%, and 28%, respectively.

Several fragmented studies were conducted in Ethiopian higher institutions, and the lifetime prevalence of GBV ranged from 35.1% (20) to 62.6% (21). Furthermore, the researchers found that a variety of attributes were associated with both the victimization and perpetration of gender-based violence in multiple studies. The following factors, for instance, were associated with a higher likelihood of gender-based violence: age, living in a rural area, having children, having witnessed family violence as a child, academic achievement, marital conflict, and partner and individual use of alcohol, tobacco products, and illegal substances (22–24). Tight family control, lack of open discussion in families on reproductive health and related personal issues, having drunken females or boyfriends, childhood witnesses of maternal abuse, as well as witnessing parental conflict, were some of the factors significantly associated with experiencing gender-based violence (20, 21, 25).

Because of the immediate morbidity and mortality associated with sexual assaults as well as the longer-term effects on women’s health, such as chronic pain, gynecological issues, sexually transmitted diseases, depression, post-traumatic stress disorder, and suicide, gender-based violence (GBV) is widely recognized as an important issue for public health (26). Despite being underreported, gender-based violence (GBV) has become common in educational institutions and hurts students’ academic performance (27).

Like any other third-world nation, there is a dearth of scientifically confirmed information on the overall magnitude or burden of gender-based violence in Africa, and Ethiopia in particular. In Ethiopia, several individual primary studies were conducted. There is only one systematic review and meta-analysis of sexual violence, including studies before 2019, but many primary studies were conducted after 2019. This study also identifies the factors that were significantly associated with GBV after identifying them from all the included primary articles. Many stakeholders are starting to create initiatives that address the fundamental root causes of GBV because they are aware of both the immediate and long-term effects of the problem in higher education institutions. So this study aimed to assess the pooled prevalence and associated factors of gender-based violence among female students in Ethiopian higher educational institutions.

### Research questions

What is the estimated pooled magnitude of gender-based violence among female students in Ethiopian higher educational institutions?

What are the estimated pooled determinants of gender-based violence among female students in Ethiopian higher educational institutions?

## Methods

### Registration and protocol

The Preferred Reporting Items for Systematic Review and Meta-Analysis (PRISMA) (**Supplementary Table 1**) guidelines were strictly followed throughout this systematic review and meta-analysis (28). It has been registered under the distinctive registration number CRD42023494760 in the International Prospective Registry of Systematic Review (PROSPERO).

### Search strategy

In this systematic review and meta-analysis, both published and unpublished studies were included to assess the pooled magnitude and associated factors of gender-based violence among female students in Ethiopian higher educational institutions. The search was conducted from 30 November to 20 December 2023. The databases used to search the primary studies were: PubMed, Health Inter-Network Access to Research Initiative, EMBASE, Google Scholar, African Journal Online, universities’ online libraries, Psychiatry Online, CINAHL, PsycINFO, and Science Direct. The following search was conducted using key terms: magnitude” OR “prevalence” OR “epidemiology OR “proportion” OR “incident” OR “burden”) AND “gender-based violence” OR “sexual violence” OR “sexual coercion” OR “sexual harassment” AND “risk factors” OR “determinants” OR “predictors” OR “correlates” AND “college students” OR “university students” “OR females” OR “students” OR “higher institution students” OR “campus” AND “Ethiopia”. Two authors (SF and TT) performed an independent search using the Boolean operators “AND” and “OR,” as appropriate, to find all key phrases.

### Eligibility criteria

#### Criteria for inclusion

This systematic review emphasized gender-based violence (sexual violence, including sexual acts through physical force, coercion, harassment, threat, or intimidation), emotional or psychological violence, and physical violence against female students in Ethiopian higher educational institutions. The primary studies that revealed the incidence of gender-based violence among female students were included in this systematic review and meta-analysis. Studies involving female students in Ethiopian colleges and universities were included in this systematic review. Articles written and reported in English were considered to be about language. Likewise, articles that had been published, regardless of their publication year limitation, were taken into consideration. This systematic review and meta-analysis allowed access to all observational study designs that reported the prevalence of gender-based violence involving female students. These include cross-sectional, comparative cross-sectional, case-control, and cohort studies.

#### Criteria for exclusion

Initially, we evaluated the titles and abstracts of the articles to determine if they were eligible. After that, we carefully read the entire text to see whether the studies applied to our review. Excluded from consideration during the article selection process were studies without an abstract or complete text. Nonetheless, we made at least two email attempts to reach the lead author before eliminating the articles. Articles that left out data regarding the prevalence of violence against female students based on gender were discarded. Furthermore, studies that included both males and females but did not report on the sexes separately were discarded.

### Data extraction

Using a standardized data extraction form that had been adopted from the Joanna Briggs Institute (JBI), the study’s two authors, SF and TT, separately extracted the data from the incorporated primary study. The two writers worked together to overcome a disagreement through discussion and resolution. The first author’s name, the year the study was published, the study area, the region in which the study was carried out, sample size, kind of educational institution (college or university), response rate, and the prevalence of gender-based violence and sexual violence among female students with 95% confidence intervals were all included in the extracted data (**Supplementary Tables 1** and **2**).

### Outcome measurements

The outcome of this study was to estimate the pooled prevalence of gender-based violence among female students of Ethiopian higher educational institutions. According to the United Nations General Assembly, gender-based violence encompasses any type of violence that results in physical, psychological, or sexual harm to the victim. This covers behaviors that can happen in both public and private settings, such as coercion and illogical denial of liberty. Gender-based violence can manifest in a variety of ways, including physical abuse such as pushing, beating, stinging, or kicking; asset destruction; using various weapons to threaten or harm her; and denial of access to health care (29, 30). This study also identifies the pooled estimates of factors associated with gender-based violence. Factors associated with gender-based violence and were expressed in odds ratios (OR).

### Quality assessment

The Joanna Briggs Institute’s (JBI) critical appraisal standard tool was initially developed to assess the methodological quality of cross-sectional studies’ prevalence. The evaluation tool has a total of nine items that were focused on the possibility of bias in study design, analysis, addressing the target population, and response rate. By calculating the mean score of the two reviewers, disagreements between the reviewers were settled. After discussion, a disagreement among the reviewers was resolved, and the third person (GN) took care of the issue. The primary studies that scored ≥ 8 out of 9 were regarded as achieving high quality; studies that scored 5–7 out of 9 were considered moderate quality; and studies that scored ≤ 4 out of 9 were considered low quality. In this study, primary studies that were achieved medium and high quality were included (**Supplementary Table 1**).

### Data synthesis and analysis

For further analysis, the Microsoft Excel spreadsheet’s extracted data was exported to STATA 14.0. Forest plots, tables, and text summaries are used to convey the findings of this systematic review and meta-analysis. The key findings and characteristics of the included studies have been explained in the summary table. STATA version 14 was used to calculate the pooled prevalence of gender-based violence, the type of GBV, the funnel plot, Eager’s test, and pooled associated factors. The I^2^ test and the forest plot graphic representation were employed to evaluate heterogeneity. We conducted subgroup analyses to investigate possible sources of heterogeneity. To verify publication bias, two techniques were applied. The first technique was the funnel plot using visual observation of the symmetry of the graph (31), whereas the second technique was Eager’s weighted regression test (32) at a 5% significant level. Due to considerable heterogeneity, we chose not to use the meta-analysis results for anything other than the overall prevalence of GBV, sexual violence, physical violence, and emotional violence. The pooled estimate of lifetime prevalence has been calculated as an overall GBV and by the type of GBV.

## Results

### Description of search results

**Figure 1:**
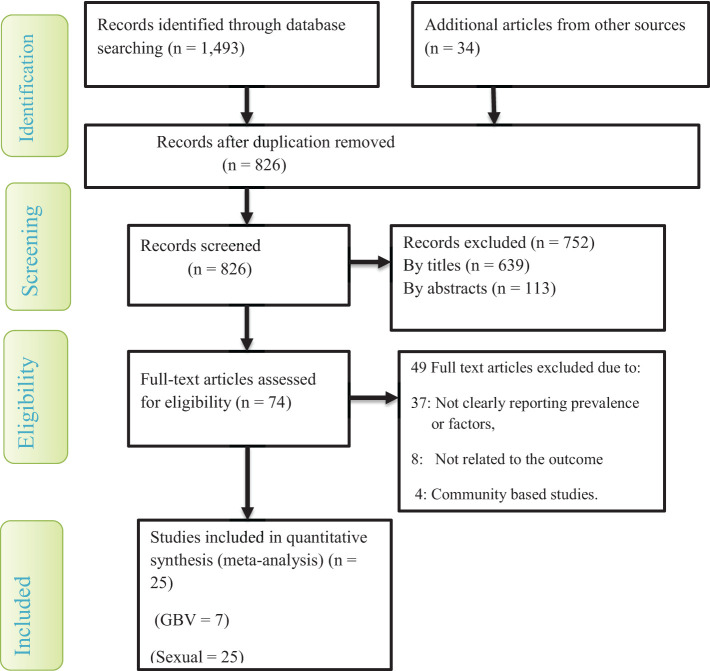
A flow chart shows study selection for a meta-analysis of gender-based violence among female students in Ethiopian higher educational institutions.

After removing duplicated articles, the search strategy resulted in 826 primary studies from various databases and other sources (**Figure 1**). Out of all the 826 primary articles searched, 752 were excluded after evaluating the titles and abstracts due to their not relying on gender-based violence. From the remaining 74 studies, 49 articles were removed after further screening because they did not provide a clear picture of the prevalence and factors that contribute to gender-based violence. Specific reasons for the 49 removed primary studies were that 37 studies did not clearly report the prevalence or factors of gender-based violence, 8 studies did not have a relation to the outcome variable, and 4 studies were community-based studies. Finally, 25 articles were reviewed for data interpretation and analysis. Out of the 25 included studies, seven determine the overall prevalence of gender-based violence, whereas most studies (n =18) exclusively investigated the prevalence of sexual violence. On the other hand, the majority of the included primary articles (n =18) were conducted in universities. Regarding the study setting, most studies (n = 8) were carried out in the Amhara regional state.

### Characteristics of the included primary studies

This study’s primary articles were conducted through a cross-sectional study design. In this systematic review and meta-analysis, three unpublished studies were included (33–35). Furthermore, the sample size of the included articles ranged from 298 to 1330, according to a study carried out in Harar (36) and Hawassa (37), respectively. In this study, all the included studies were carried out between 2004 and 2022 and available online between 2004 and 2023. The total number of respondents in this review was 13,013. Regarding the assessment tools, except for two primary studies (20, 38), all other included studies were assessed using the WHO Multi-Country Study of Violence Against Women. Eight studies were conducted in the South Nations Nationalities and Peoples (SNNPs) regional state, while seven studies were carried out in the Amhara regional state. Furthermore, six studies were conducted in the Oromia regional state. The other four single studies were conducted in Tigray, Harari, Somali regional states, and Addis Ababa city. Fourteen of the twenty-five studies were conducted in universities; the other eleven studies were in colleges (**Tables 1**, **2**). All the primary articles included used a random sampling technique and specified the target population. Regarding the quality of the included studies, fifteen (60%) of them achieved high quality, whereas the remaining ten (40%) of them scored medium quality.

**Table 1:** Characteristics of included studies on the prevalence of gender-based violence among female students in Ethiopian higher educational institutions.

Authors with year of publication	Study year	Place	Response rate (%)	Sample size	Lifetime GBV (%)	12 month GBV (%)	GBV after Joining institution (%)	Lifetime sexual violence (%)	Lifetime physical violence (%)
Gebrie et al. (2022) (21)	2021	Dessie	98.7	401	62.6	N/A	N/A	39.20	62.30
Negero et al. (2019) (33)	2016	Debremarkos	91	766	36	N/A	N/A	18.9	17.1
Birkie et al. (2020) (20)	2018	Gondar	92.2	299	35.1	N/A	N/A	34.1	29.5
Abubeker et al. (2021) (36)	2016	Harar	98.7	298	57.7	N/A	N/A	46.6	36.2
Arnold et al. (2008) (37)	2006	Hawassa	100	1330	59.9	40.3	46.1	54.9	18.5
Yaynshet G (2007) (34)	2007	Mekelle	96.2	1024	62.1	40.2	N/A	45.4	46.3
Workye et al. (2023) (39)	2021	Wolkite	100	353	46.2	18.7	28	47	56.1

**Table 2:** Characteristics of included studies on the prevalence of sexual violence among female students in Ethiopian higher educational institutions.

Authors	Study year	Place	Response rate (%)	Sample size	Lifetime sexual violence (%)	12 month sexual violence (%)	Sexual violence after joining institution (%)
Gebrie et al.(2022) (21)	2021	Dessie	98.7	401	39.20	N/A	N/A
Negero et al.(2019) (33)	2016	Debremarkos	91	766	18.9	N/A	N/A
Birkie et al.(2020) (20)	2018	Gondar	92.2	299	34.1	N/A	N/A
Abubeker et al.(2021) (36)	2016	Harar	98.7	298	46.6	N/A	N/A
Arnold et al.(2008) (37)	2006	Hawassa	100	1330	54.9	40.3	46.1
Yaynshet G (2007) (34)	2007	Mekelle	96.2	1024	45.4	40.2	N/A
Workye et al.(2023) (39)	2021	Wolkite	100	353	47	18.7	28
Benti and Teferi (2015) (40)	2013	Nekemte	99.6	562	41.3	31.9	N/A
Bekele and Deressa (2014) (25)	2012	Ambo	98.8	590	76.4	43.7	N/A
Shimekaw et al.(2013) (41)	2012	Bahirdar	99.1	536	37.3	N/A	N/A
Temesgan W.Z. et al.(2021) (42)	2019	Debremarkos	97.8	413	27.1	17.2	21.3
Mamaru A et al.(2015) (43)	2014	Jimma	N/A	385	78.2	N/A	N/A
Tegegne KT et al. (2019) (44)	2018	Hawassa	100	385	38.4	N/A	N/A
Esayas et al.(2023) (45)	2020	Hawassa	95.6	330	61.2	61.2	N/A
Seid A (2018) (46)	2018	Jigjiga	94.3	561	36.5	17	17
A Takele and T Setegn (2014) (38)	2012	Madawalabo	96.6	397	41.1	25.4	N/A
SM Hassen and BH Mohammed (2021) (47)	2016	Debrebirhan	91.5	627	54.9	N/A	35.6
Tora A (2013) (48)	2011	Wolaita Sodo	N/A	374	23.4	N/A	N/A
Kassa S et al.(2019) (49)	2018	Dessie	97.8	402	59.7	34.1	N/A
Bekele et al.(2015) (50)	2014	Madawalabo	97.9	605	36.5	36.5	N/A
Yemsrach et al.(2017) (51)	2016	Bishoftu	100	395	43.3	N/A	N/A
EG Sendo and M Meleku (2015) (52)	2013	Hawassa	100	336	42.9	3	14.3
Henock et al.(2015) (53)	2013	Mizan Tepi	94.4	570	75.4	63	66.3
Adinew and Hagos (2017) (27)	2015	Wolaita Sodo	95.4	462	45.4	24.4	36.1
Tadesse S (2004) (54)	2004	Addis Ababa	N/A	612	58	41.8	N/A

### The pooled prevalence of gender-based violence among female students in Ethiopian higher educational institutions

**Figure 2:**
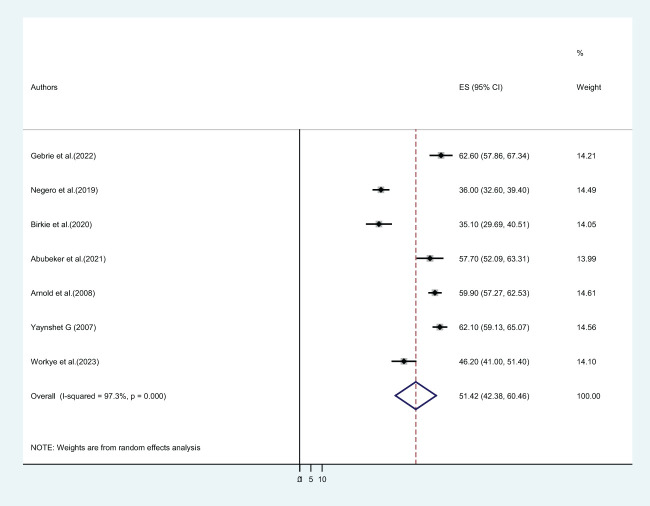
The pooled lifetime prevalence of gender-based violence among female students in Ethiopian higher educational institutions.

A total of 4,471 participants from seven studies were reviewed. The result shows the pooled prevalence of gender-based violence among female students in Ethiopian higher educational institutions was found to be 51.42% (CI: 42.38, 60.46) (**Figure 2**). Only three studies reported the twelve-month or one-year prevalence of GBV. Based on our findings, the pooled twelve-month prevalence of gender-based violence was 33.16% (21.06, 45.26). Furthermore, two primary studies reported the prevalence of gender-based violence after students joined the institution.

### The pooled prevalence of sexual violence among female students in Ethiopian higher educational institutions

**Figure 3:**
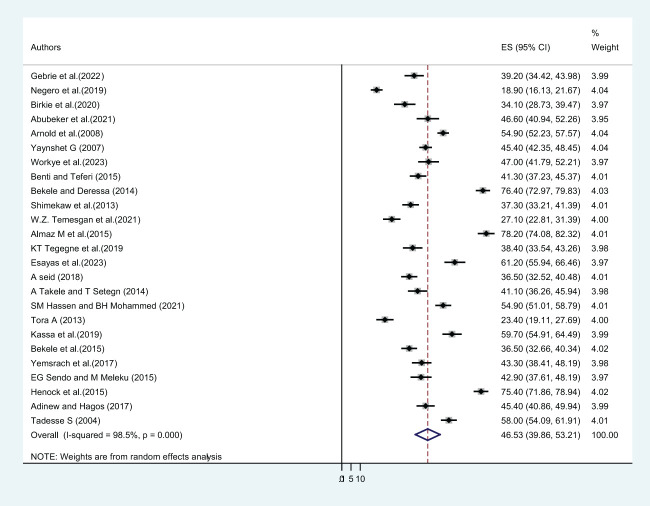
The pooled prevalence of lifetime sexual violence among female students in Ethiopian higher educational institutions.

**Figure 4:**
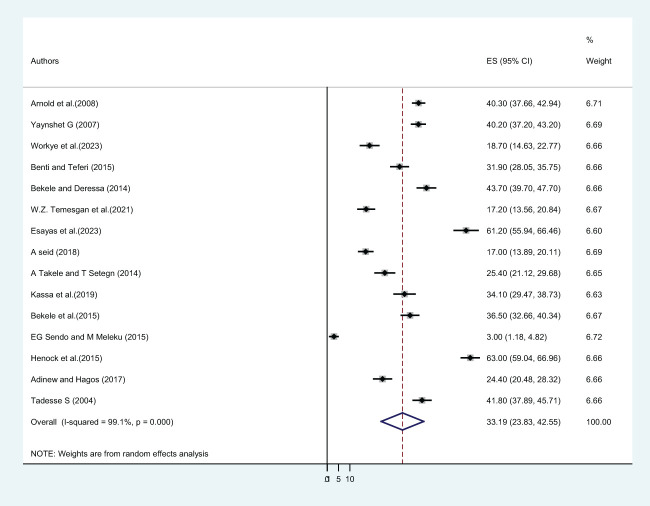
The twelve month pooled prevalence of sexual violence among female students in Ethiopian higher educational institutions.

**Figure 5:**
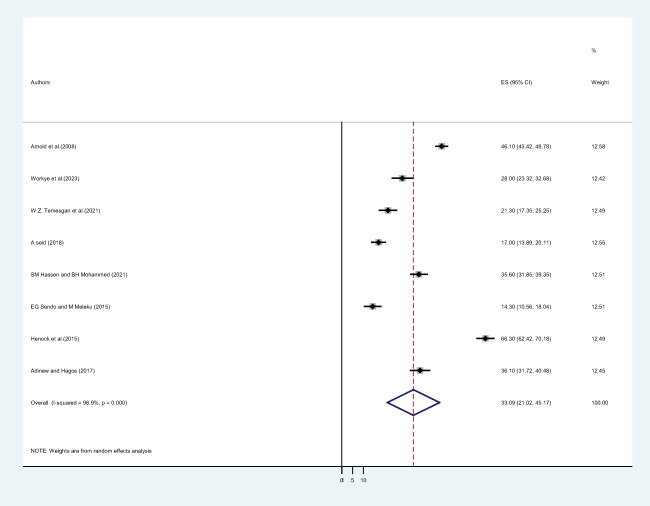
The pooled prevalence of sexual violence among female students in Ethiopian higher educational institutions after joining the institution.

In this study, twenty-five primary studies with a total of 13,013 female students were included. The pooled lifetime prevalence of sexual violence among female students in Ethiopian higher educational institutions was 46.53% (CI: 39.86, 53.21) (**Figure 3**). Out of twenty-five included primary studies, fifteen articles reported the twelve-month prevalence of sexual violence. The pooled twelve-month/one-year prevalence of sexual violence among 8,547 female students was 33.19% (CI: 23.83, 42.55) (**Figure 4**), whereas only eight included articles reported the prevalence of sexual violence after students joined a college or university. The pooled prevalence of sexual violence among 4,652 female higher educational institution students was found to be 33.09% (21.02, 45.17) (**Figure 5**).

### The pooled lifetime prevalence of physical violence among female students in Ethiopian higher educational institutions

**Figure 6:**
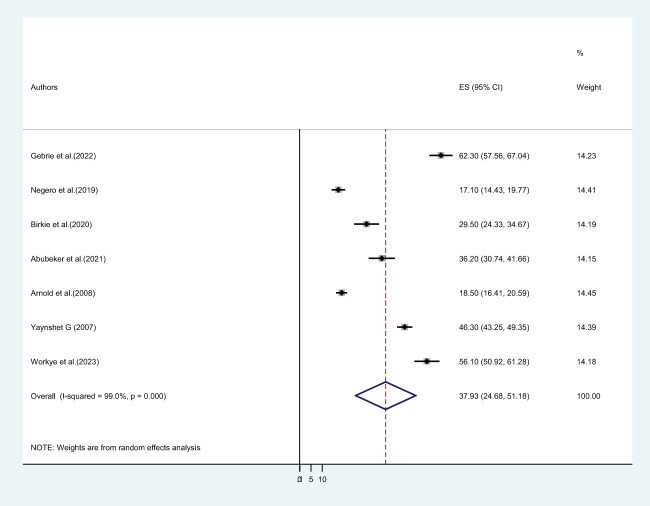
The pooled prevalence of lifetime physical violence among female students in Ethiopian higher educational institutions.

In this systematic review and meta-analysis, seven primary studies reported the lifetime prevalence of physical violence. Our finding revealed that the pooled lifetime prevalence of physical violence among Ethiopian higher educational institution female students was 37.93% (CI: 24.68, 51.18) (**Figure 6**).

### Heterogeneity and publication bias

To assess the statistical heterogeneity of the included studies, the statistics (I^2^) test was applied. We found that there was a high degree of heterogeneity between studies, as evidenced in (I^2^ = 97.3%, p = 0.000), (I^2^ = 99.0%, p = 0.000), and (I^2^ = 98.5%, p = 0.000) for lifetime gender-based violence, sexual violence, and physical violence, respectively.

**Figure 7:**
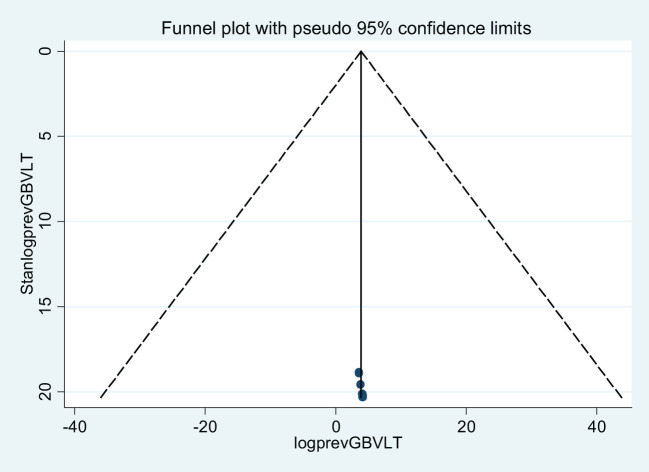
A funnel plot of lifetime gender-based violence among female students in Ethiopian higher educational institutions.

**Figure 8:**
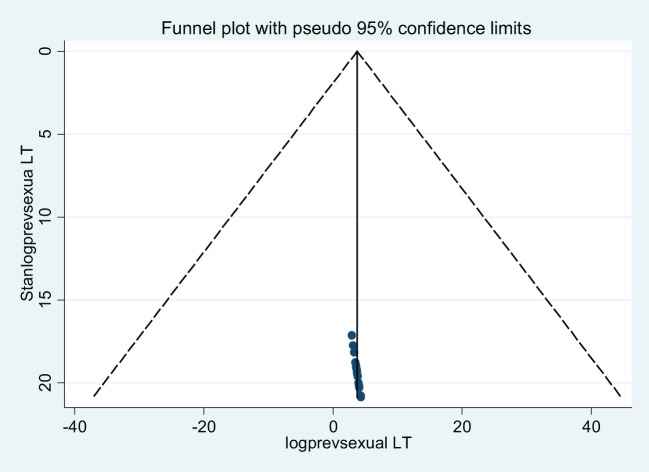
A funnel plot of lifetime sexual violence among female students in Ethiopian higher educational institutions.

Concerning publication bias, we have applied two techniques. The first technique was a funnel plot, which shows a symmetric distribution of visual observation (**Figures 7**, **8**) for lifetime GBV and sexual violence, whereas the second technique was Egger’s test. As expressed in tables (**Tables 3**, **4**), we confirmed that there was no publication bias because Egger’s test bias level was greater than the significance (>0.05) (p-value = 0.439 and 0.902) for lifetime gender-based violence and sexual violence.

**Table 3:** Egger’s test of lifetime gender-based violence among female students in Ethiopian higher educational institutions.

Std_Eff	Coef.	Std. Err.	t	P>|t|	[95% Conf. Interval]
**Slope**	66.37392	15.94594	4.16	0.009	25.38358 107.3642
**Bias**	-6.953667	8.28115	-0.84	0.439	-28.24104 14.33371

**Table 4:** Egger’s test of lifetime sexual violence among female students in Ethiopian higher educational institutions.

Std_Eff	Coef.	Std. Err.	t	P>|t|	[95% Conf. Interval]
**Slope**	48.84627	16.65233	2.93	0.007	14.3983 83.29424
**Bias**	-1.000936	8.073638	-0.12	0.902	-17.70253 15.70066

### Sub-group analysis

Since we observed a high level of heterogeneity between the included primary articles, we have conducted a sub-group analysis using sub-groups. These sub-groups are based on the regional states where the study was conducted, the type of institution, and the study year. The prevalence of lifetime gender-based violence in two regional states (Harari and Tigray) was higher than in studies carried out in SNNPs and the Amhara regional states (60.57%, 53.24%, and 44.56%, respectively). Regarding the type of institution, the studies conducted found that the lifetime prevalence of GBV among college students was higher than among university students (55.66% and 40.90%, respectively). Furthermore, the pooled prevalence of lifetime GBV among studies conducted before 2017 was higher than in studies conducted after 2017 (**Table 5**). On the other hand, the lifetime prevalence of sexual violence in the Oromia regional state (52.82%) was higher than the SNNPs (48.60%) and Amhara regional states (38.71%). The lifetime prevalence of sexual violence among university students was higher than among college students, in contrast to GBV, at 49.60% and 42.63%, respectively. The lifetime prevalence of sexual violence in studies conducted before 2017 was higher than in studies conducted after 2017 (**Table 6**).

**Table 5:** Subgroup analysis of lifetime gender-based violence among female students in Ethiopian higher educational institutions.

Variables	Sub-groups	Number of studies	Prevalence (95% CI)	I^2^(%)	P-value
Regions	Amhara	3	44.56(27.27, 61.84)	97.8	0.000
	SNNPs	2	53.24(39.82, 66.66)	95.3	0.000
	Others	2	60.57(56.46, 64.68)	45.8	0.174
Type of institutions	University	2	40.90(30.91, 50.89)	95.0	0.000
	College	5	55.66(47.76, 63.55)	95.1	0.000
Study year	Before 2017	4	53.92(41.72, 66.11)	98.1	0.000
	After 2017	3	48.01(32.11, 63.91)	96.6	0.000

Others: Tigray and Harari regional states.

SNNPs: Southern Nation Nationalities and Peoples.

**Table 6:** Subgroup analysis of lifetime sexual violence among female students in Ethiopian higher educational institutions.

Variables	Sub-groups	Number of studies	Prevalence (95% CI)	I^2^(%)	P-value
Regions	Amhara	7	38.71(26.87, 50.55)	98.3	0.000
	Oromia	6	52.82(36.70, 68.95)	98.9	0.000
	SNNPs	8	48.60(37.25, 59.96)	98.3	0.000
	Others	4	46.62(37.85, 55.40)	94.8	0.000
Type of institutions	University	14	49.60(38.51, 60.69)	95.0	0.000
	College	11	42.63(36.98, 48.29)	99.1	0.000
Study year	Before 2017	17	48.24(39.54, 56.94)	98.9	0.000
	After 2017	8	42.86(34.50, 51.23)	95.9	0.000

Others: Tigray, Addis Ababa, Somali, and Harari regional states.

SNNPs: Southern Nation Nationalities and Peoples.

### Sensitivity analysis

A sensitivity analysis was carried out to evaluate the heterogeneity between the reviewed articles. To examine this heterogeneity, we have applied to remove one study to determine the effect of each study’s results on the pooled magnitude of lifetime GBV and sexual violence. Our finding showed that all of the results relied on the expected 95% CI of the pooled prevalence of GBV and sexual violence (**Tables 7**, **8**). So we concluded that in this study, the exclusion of a single study didn’t affect the pooled magnitude of both GBV and sexual violence.

**Table 7:** Sensitivity analysis of lifetime gender-based violence among female students in Ethiopian higher educational institutions.

Authors	Estimate prevalence (95% CI)	Heterogeneity	
		I^2^(%)	p-value
Gebrie et al.(2022) (21)	49.56(39.49, 59.63)	97.6	0.000
Negero et al.(2019) (33)	54.10(46.67, 61.54)	95.0	0.000
Birkie et al.(2020) (20)	54.09(44.97, 63.20)	97.2	0.000
Abubeker et al.(2021) (36)	50.39(40.21, 60.57)	97.8	0.000
Arnold et al.(2008) (37)	49.96(39.07, 60.86)	97.4	0.000
Yaynshet G(2007) (34)	49.60(39.27, 59.92)	97.3	0.000
Workye et al.(2023) (39)	52.27(42.15, 62.40)	97.7	0.000

**Table 8:** Sensitivity analysis of lifetime sexual violence among female students in Ethiopian higher educational institutions.

Authors	Estimate prevalence (95% CI)	Heterogeneity	
		I^2^(%)	p-value
Gebrie et al.(2022) (21)	46.84(39.94, 53.74)	98.6	0.000
Negero et al.(2019) (33)	47.70(41.58, 53.82)	98.1	0.000
Birkie et al.(2020) (20)	47.05(40.19, 53.90)	98.6	0.000
Abubeker et al.(2021) (36)	46.53(39.64, 53.42)	98.6	0.000
Arnold et al.(2008) (37)	46.18(39.18, 53.24)	98.5	0.000
Yaynshet G (2007) (34)	46.58(39.51, 53.65)	98.6	0.000
Workye et al.(2023) (39)	46.51(39.61, 53.42)	98.6	0.000
Benti and Teferi (2015) (40)	46.75(39.80, 53.70)	98.6	0.000
Bekele and Deressa (2014) (25)	45.28(38.95, 51.61)	98.3	0.000
Shimekaw et al.(2013) (41)	46.92(40.00, 53.83)	98.6	0.000
Temesgan W.Z et al.(2021) (42)	47.34(40.58, 54.11)	98.5	0.000
Mamaru A et al.(2015) (43)	45.21(38.77, 51.66)	98.3	0.000
Tegegne KT et al.(2019) (44)	46.87(39.98, 53.76)	98.6	0.000
Esayas et al.(2023) (45)	45.93(39.10, 52.77)	98.6	0.000
Seid A(2018) (35)	46.95(40.04,53.86)	98.6	0.000
A Takele and T Setegn (2014) (38)	46.76(39.85, 53.67)	98.6	0.000
SM Hassen and BH Mohammed (2021) (47)	46.18(39.24, 53.12)	98.6	0.000
Tora A (2013) (48)	47.50(40.81, 54.19)	98.5	0.000
Kassa S et al.(2019) (49)	45.99(39.13, 52.85)	98.6	0.000
Bekele et al.(2015) (50)	46.95(40.03, 53.87)	98.6	0.000
Yemsrach et al.(2017) (51)	46.67(39.75, 53.58)	98.6	0.000
EG Sendo and M Meleku (2015) (52)	46.68(39.78, 53.58)	98.6	0.000
Henock et al.(2015) (53)	45.32(38.91, 51.74)	98.3	0.000
Adinew and Hagos (2017) (27)	46.58(39.65, 53.51)	98.6	0.000
Tadesse S (2004) (54)	46.05(39.15, 52.96)	98.6	0.000

### Factors associated with lifetime gender-based violence among female students in Ethiopian higher educational institutions

**Figure 9:**
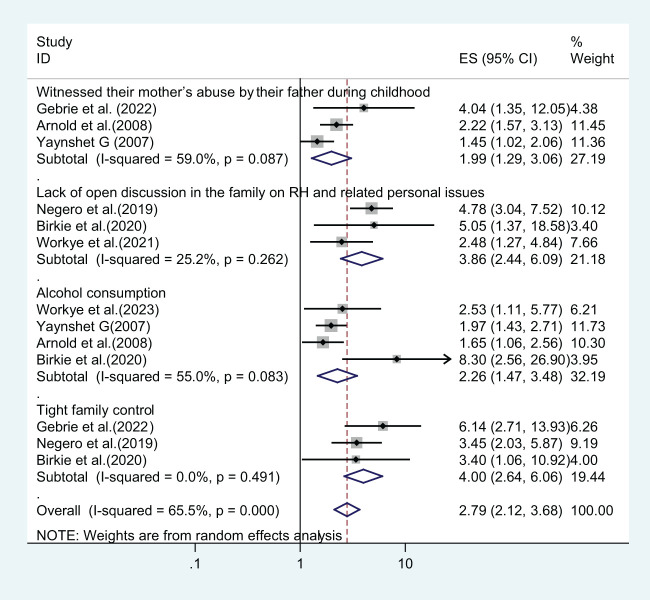
Factors associated with lifetime gender-based violence among female students in Ethiopian higher educational institutions.

From the reviewed primary articles, several factors were significantly associated with lifetime gender-based violence among Ethiopian higher institution female students. For example, witnessing their mother’s abuse by their father during childhood, a lack of open discussion in the family on reproductive health and related issues, and having tight family control or a strict parenting style over the girl’s behavior were factors significantly associated with GBV in three primary studies. Furthermore, alcohol consumption was significantly associated with GBV in four primary studies. This systematic review and meta-analysis revealed that those who witnessed their mother’s abuse by their father during childhood were almost two times more likely to suffer from gender-based violence than their counterparts (AOR = 1.99, CI: 1.29, 3.06). Female students who lack open discussion in the family on reproductive health and related personal issues were approximately four times more likely to suffer from GBV compared to students who have open discussions with their family (AOR = 3.86, CI: 2.44, 6.09). Respondents who have alcohol drinking habits had more than two times higher odds of being exposed to GBV (AOR = 2.26 CI: 1.47, 3.48), while those who have tight family control or a strict parenting style were four times more experienced with GBV (AOR = 4.00 CI: 2.64, 6.06) (**Figure 9**).

### Factors associated with lifetime sexual violence among female students in Ethiopian higher educational institutions

In the included primary studies and this systematic review and meta-analysis, four factors were significantly associated with sexual violence. Female students who had drunken friends, two or more sexual partners, a regular boyfriend, and chat chewing were significantly associated with sexual violence.

**Figure 10:**
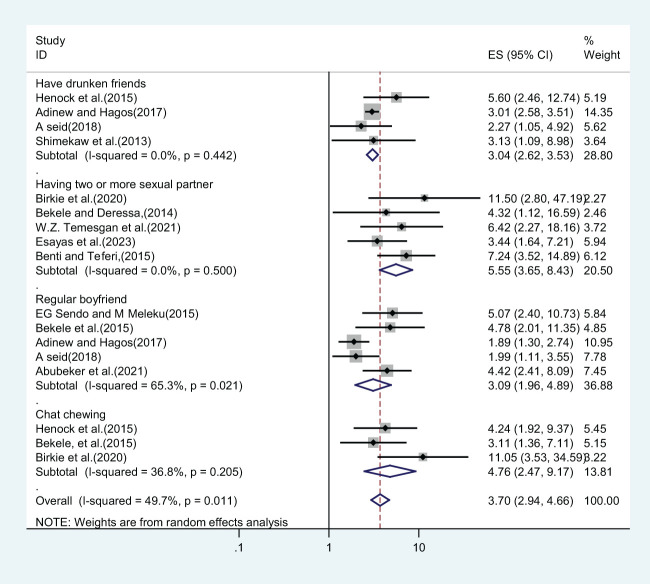
Factors associated with lifetime sexual violence among female students in Ethiopian higher educational institutions.

Respondents who had drunken friends and regular boyfriends were three times more exposed to sexual violence compared to non-drunken friends (AOR = 3.04, CI: 2.62, 3.53) and not having regular boyfriends (AOR = 3.09, 1.96, 4.89), respectively. Female students having two or more sexual partners were more than five times more likely to suffer from sexual violence in their lives compared to their counterparts (AOR = 5.55, CI: 3.65, 8.43). On the other hand, students who were chewing chat were approximately five times more exposed to sexual violence compared to non-chewers (AOR = 4.75, CI: 2.47, 9.17) (**Figure 10**).

## Discussion

This study aimed to assess the pooled magnitude and factors significantly associated with gender-based violence among female students in Ethiopian higher educational institutions. It incorporates a total of 4,471 and 13,013 female university and college students in the final review for GBV and sexual violence, respectively. This systematic review and meta-analysis was also the first of its kind in Ethiopia to show aggregated information on gender-based violence in this population. Our finding revealed that the lifetime prevalence of gender-based violence among female students of Ethiopian higher educational institutions was found to be 51.42% (CI: 42.38, 60.46) (**Figure 2**). Three studies reported the twelve-month or one-year prevalence of GBV, and the pooled twelve-month magnitude of gender-based violence was 33.16% (21.06, 45.26).

This study indicated that the burden of gender-based violence is very high because it affects more than half of female students. The overall rate of GBV in Ethiopian educational institutions is still extremely high at this moment in time. Since the age of schooling is a way to form unions, people are more likely to face relationship stressors that might lead to gender-based violence (55). The pooled prevalence of lifetime gender-based violence was consistent with other systematic review findings carried out in Ethiopia among high school students (56) and three Sub-Saharan African countries (14).

On the other hand, this finding was higher than a community study conducted in five Asian countries among the general population (57). Because students at higher educational institutions want to receive good grades, they might start relationships with teachers and students, which might result in violence. Understanding someone’s typical behavior will enable you to recognize even minor behavioral variations between him or her and act as a warning sign that anything is disastrous (58). The other possible reason might be related to the difference in the availability of information on sexual and reproductive health issues. Because they live in new communities and environments, female students at higher education institutions may not have the opportunity to initiate a discussion. While some of the variations may be explained as discrepancies in data collection techniques and cultural variations that may affect self-reporting, the region’s noticeably more traditional family and social structures are probably major contributing causes (59).

This systematic review and meta-analysis also determine the lifetime prevalence of sexual violence. About twenty-five primary studies with a total of 13,013 female students were included. The pooled lifetime prevalence of sexual violence among female students in Ethiopian higher educational institutions was 46.53% (CI: 39.86, 53.21). Out of twenty-five included primary studies, fifteen articles reported the twelve-month prevalence of sexual violence, and the pooled twelve-month/one-year prevalence of sexual violence among 8,547 female students was 33.19% (CI: 23.83, 42.55).

On the other hand, the frequency of sexual violence against students who are enrolled in college or university is only addressed in eight of the included articles. A total of 4,652 female students attending higher education institutions had a pooled prevalence of sexual violence of 33.09% (21.02, 45.17). Sexual violence is one type of relationship-based violence that tends to be committed by close friends and family members (29). It is one of the parameters for gender-based violence that can be used to evaluate how well the sustainable development goal is succeeding. Sexual violence has serious and numerous negative effects on women’s health. As the victim draws herself, it causes profound psychological alterations in her interactions with those in her immediate social circle and the community at large. As such, it leaves a long-lasting bad impression on how the victim views herself, the situation, and others (60). Victims of sexual violence were more likely than non-victims to have five or more days of poor mental health (61). Following sexual violence, treatment should focus on both the protective effects of behavioral factors like exercise, a healthy diet, and quitting smoking, as well as risk factors like low income, low educational attainment, and a lack of emotional support. It is necessary to have comprehensive, integrated models of care that address the social, mental, and physical effects of sexual violence.

This finding was in line with a study conducted in Ethiopia among higher institution female students that reviewed ten primary studies (62). In contrast, the pooled prevalence of lifetime sexual violence in this study was lower than the other systematic review and meta-analysis conducted among high school and university students in Ethiopia (63). This comparison is based on a sub-group analysis that found the lifetime prevalence of sexual violence among studies conducted on college and university students was 53.76%. In this study, the included studies were twenty-five in number, whereas the included studies in Mekonnen et al. (2022) were only seven.

Furthermore, the pooled prevalence of lifetime sexual violence in this review was about 29% and 20% higher than a systematic review and meta-analysis conducted in three sub-Saharan African countries and other sixteen countries (13, 14). This significant variation can result from varying samples as well as the definitions and methods of assessment employed in different studies on sexual violence. Most studies used a single item for estimating sexual violence. This could have resulted in either an overestimation or an underestimation of the incidence of sexual violence. This finding is even two times higher than that of females in higher humanitarian emergencies (64).

In this systematic review and meta-analysis, seven primary studies reported the lifetime prevalence of physical violence. Our finding revealed that almost four out of ten female students at higher educational institutions suffer from physical violence throughout their lives (**Figure 6**). The pooled magnitude of lifetime physical violence in this study was more than twofold compared to a meta-analysis and systematic review carried out in three Sub-Saharan African countries (14). Compared to other regions, this data shows that students in Ethiopia suffer from violence on an ongoing basis. These differences can have resulted from definitions and techniques for assessing physical violence, as well as from social, cultural, and normative factors. An alternative explanation could be that women in developing countries rationalize the acceptance of men abusing their partners; this is a common occurrence in Ethiopia (65, 66). Furthermore, most of these systematic reviews made use of their self-reporting instruments, and most research employed different definitions of physical violence and different sample sizes to evaluate prevalence.

Considering the significant degree of heterogeneity we found across the primary papers we investigated, we used sub-groups to perform a sub-group analysis. These subgroups are determined using the institution type, study year, and the regional states in which the study was carried out. Compared to research conducted in SNNPs and the Amhara regional states, the prevalence of lifetime gender-based violence was higher in two regional states (Harari and Tigray) (60.57%, 53.24%, and 44.56%, respectively).

According to the studies, college students had a greater lifetime prevalence of GBV than university students (55.66% and 40.90%, respectively). Moreover, research conducted before 2017 had a greater pooled prevalence of lifetime GBV than that conducted after 2017 (**Table 5**). During the year 2017, Ethiopia revised and implemented the adopted international and regional instruments, like the African Charter on the Rights of the Child, the Convention on the Elimination of Discrimination Against Women, and the Convention on the Rights of the Child. The creation of child, disability, and gender-sensitive educational facilities, as well as the provision of “safe, non-violent, inclusive, and effective learning environments for all,” are among the most recent targets included in the Sustainable Development Goals for Education.

Compared to the SNNPs (48.60%) and Amhara regional states (38.71%), the Oromia regional state had a greater lifetime prevalence of sexual violence (52.82%). In contrast to GBV, the lifetime prevalence of sexual violence was greater among university students than among college students, with a prevalence of 49.60% and 42.63%, respectively. Due to their lifestyle, university students may not have parental supervision or protection. Living on campus, they are more emotionally connected to other students and staff than they are to their parents. Due to this state, university students may be more vulnerable to sexual violence by peers, instructors, or staff members compared to college students. Research conducted before 2017 found a greater lifetime frequency of sexual violence than research conducted after 2017 (**Table 6**).

This systematic review also identified several contributing variables associated with lifetime gender-based violence and sexual violence. Witnessing their mother’s abuse by their father during childhood, a lack of open discussion in the family about reproductive health and related personal issues, alcohol consumption, and tight family control were some of the factors significantly associated with lifetime gender-based violence (**Figure 9**). Furthermore, those who had drunken friends, a regular boyfriend, two or more sexual partners, and chat chewing were factors significantly associated with lifetime sexual violence (**Figure 10**).

Compared to other students, female students who witnessed their father abuse their mother during childhood were nearly twice as likely to experience gender-based violence (AOR = 1.99, CI: 1.29, 3.06). This finding is consistent with studies conducted in three African countries (14). A systematic review in Ethiopia reported that approximately one-third (27%) of husbands abuse or experience domestic violence toward their wives or partners without clear reasons (67), and this is adopted as a normal culture. So students might not count it as violence in their lives. This suggests that a girl may have adapted to and come to accept this harmful role from the beginning of her life.

Female students who were not able to openly discuss reproductive health and related personal issues within their family were approximately four times more likely to suffer from GBV compared to students who were able to discuss assorted topics with their family (AOR = 3.86, CI: 2.44, 6.09). The reason for this could be that families serve as a bridge between individuals and society at large. For this reason, it is critical to support and defend women’s rights to control and make free decisions about matters related to their sexuality and to be able to defend norms related to consent and sexual violence (68).

The likelihood of experiencing lifetime GBV was more than two times (AOR = 2.26, CI: 1.47, 3.48) more likely among respondents who have alcohol drinking habits than their counterparts. This finding is consistent with a previous study in Ethiopia, Sub-Saharan African countries (14, 62), and high-income countries (69). This could be because alcohol use can lower people’s level of consciousness, problem-solving skills, and judgment, and weaken their ability to defend themselves against abuse, leaving them open to rape or other unwanted and inappropriate sexual contact (70, 71). Additionally, those who have tight family control or a strict parenting style were four times more likely to suffer from lifetime GBV (AOR = 4.00, CI: 2.64, 6.06). The possible explanation for this could be that a typical parenting approach gives guidance along with the independence to make personal decisions and can strengthen self-leadership confidence. The other reason could be that young females are more likely to test something new, which raises the prospect of GBV. “Students who live with their families are closely monitored, but when they attempt to manage themselves, particularly those from rural areas, they are exposed to various negative behaviors that put them at risk for gender-based violence.”

Regarding sexual violence, respondents who had drunken friends were three times more likely to suffer from sexual violence in comparison to those who did not have drunken friends (AOR = 3.04, CI: 2.62, 3.53). Individuals who have drunken friends are easily vulnerable to drinking alcohol together with them due to peer pressure, and this might increase the male perpetration rate (72). Additionally, it has been alleged that male students purchase alcoholic drinks for female students, forcing them to consume more than they should and assaulting them sexually while they are intoxicated (73).

Students who had regular boyfriends were three times more prone to sexual violence compared to those who did not have regular boyfriends (AOR = 3.09, 1.96, 4.89). Girls who have boyfriends are more likely to be sexually abused because they trust their friends and spend a lot of time with them in dangerous settings, including drug homes, couple houses, and nightclubs. Various studies have demonstrated that intimate partners, such as boyfriends or husbands, are the ones who commit sexual violence most commonly (74). The other possible explanation is that females with boyfriends are less inclined to refuse requests out of concern that their friendships might be going to end. Furthermore, classmates may have encouraged students with boyfriends to become victims of sexual violence.

The likelihood of experiencing sexual violence was more than five times higher among female students who had multiple sexual partners compared to their counterparts (AOR = 5.55, CI: 3.65, 8.43). A study in Ethiopia indicated that nearly half (45%) of college students have multiple sexual partners in their lives (75). Having multiple sexual partners increases the likelihood of being exposed to conflicts between men and coercive partners (76). Limiting sexual partners is an effective violence prevention approach that needs cooperation within a partnership (77, 78). On the other hand, students who were chewing chat were approximately five times more prone to sexual violence compared to non-chewers (AOR = 4.75, CI: 2.47, 9.17). Any habit, including chat-chewing, has a direct impact on psychological and mental processes that lower self-esteem and control, making people less able to negotiate a peaceful settlement of disputes in relationships. Healthcare assistance and forensic sciences together create a comprehensive support system for victims of violence, addressing their medical and psychological needs while also contributing to the pursuit of justice. The collaboration between healthcare providers and forensic experts ensures that victims receive holistic care and that perpetrators are held accountable. Immediate medical care is essential to treat injuries, prevent infections, and manage pain. This includes emergency care for life-threatening conditions, suturing wounds, setting fractures, and providing necessary medications (79). Victims of violence often suffer from psychological trauma. Immediate and ongoing psychological support, including counseling and therapy, helps in addressing acute stress reactions and preventing long-term mental health issues such as PTSD, anxiety, and depression (80).

Forensic sciences play a significant role in this context by providing the necessary tools and methods to document, analyze, and interpret injuries and evidence related to violence. Forensic experts meticulously document injuries and collect physical evidence, which is critical for legal proceedings (81). Proper documentation can include photographs, detailed injury reports, and preservation of biological samples. They can help in identifying patterns of abuse, which may be useful in cases involving serial offenders or systematic abuse. This can aid law enforcement in linking cases and identifying perpetrators. Forensic professionals often provide training to healthcare providers, law enforcement, and legal professionals on best practices for handling, documenting, and processing evidence related to violence. This enhances the overall response to violence against individuals (82, 83).

### Strengths and limitations of the study

This systematic review and meta-analysis strictly followed the Preferred Reporting Items for Systematic Reviews and Meta-Analyses (PRISMA) protocols. In this study, authors have searched primary articles through numerous databases, and to reduce the biases of reviewers and their assessment of the quality of the papers that they evaluated, the third author solved the discrepancy. This review also addressed the discrepancy based on the sub-group analysis through the regional state of the study conducted, the type of institution, and the study year. We have also figured out the contributing factors to gender-based violence and sexual violence. Even though this review has strengths, it also has some limitations. There was a significant level of heterogeneity between the included primary studies. All the included primary studies were conducted through an institutional-based cross-sectional study design, which only illustrates a temporal relationship rather than a true cause-and-effect relationship. Almost all reviewed primary studies were assessed using the WHO Multi-Country Study of Violence Against Women, which may underestimate or overestimate the prevalence of gender-based violence and sexual violence.

### Implications of the study

Despite the significant heterogeneity throughout the studies, GBV remains a noteworthy public health issue. This indicates that GBV is prevalent among adolescents attending higher educational institutions in Ethiopia. This study has implications for the building blocks of preventative measures, policies, and implementation strategies in Ethiopia. It also implies that Ethiopia needs to implement intervention techniques, a policy to avoid adolescent violence, and address service offerings. GBV impairs the achievement of the Sustainable Development Goals (SDGs) and hurts young people, particularly women and girls. Eliminating GBV in educational institutions and the community by teaching the public, parents, and students about the prevalence, causes, and effects of the issue is essential to achieving Sustainable Development Goal 5 (gender equality).

### Conclusions and recommendations

This systematic review and meta-analysis showed that half the higher-institutional female students had experienced gender-based violence in Ethiopia. Witnessing their mother’s abuse by their father during childhood, a lack of open discussion in the family about reproductive health and related personal issues, alcohol consumption, and tight family control were some of the factors significantly associated with lifetime gender-based violence. Additionally, those who had drunken friends, a regular boyfriend, two or more sexual partners, and chat chewing were factors significantly associated with lifetime sexual violence. The federal and regional governments, educational institutions, and relevant stakeholders should develop and implement effective educational institution-based interventions to address violence against female students. The implementation of laws and interventions that were focused on the significant factors was crucial.

## Data Availability

The original contributions presented in the study are included in the article/**Supplementary Material**. Further inquiries can be directed to the corresponding author.
