# Supplementary material for: Magnitude and determinants of gender-based violence among female students in Ethiopian higher educational institutions: a systematic review and meta-analysis
Source: Front Psychiatry. 2024 Aug 22;15:1387032. doi: 10.3389/fpsyt.2024.1387032 (PMC11374738; doi:10.3389/fpsyt.2024.1387032)
Supplement: Supplementary file 4 [file Table2.docx]

Supplementary table 2: Quality assessment of gender-based violence among female students in Ethiopian higher educational institutions that were included studies in this meta-analysis and systematic review.

| Authors | Q1 | Q2 | Q3 | Q4 | Q5 | Q6 | Q7 | Q8 | Q9 | Total score (9) |
| --- | --- | --- | --- | --- | --- | --- | --- | --- | --- | --- |
| Gebrie et al.(2022)(21) | Y | Y | Y | Y | Y | Y | Y | Y | Y | 9 |
| Negero et al.(2019)(34) | Y | Y | Y | NA | Y | Y | NA | NA | Y | 6 |
| Birkie et al.(2020)(20) | Y | Y | NA | Y | Y | Y | Y | Y | Y | 8 |
| Abubeker et al.(2021)(37) | Y | Y | NA | Y | NA | Y | Y | Y | Y | 7 |
| Arnold et al.(2008)(38) | Y | Y | Y | Y | Y | Y | Y | Y | Y | 9 |
| Yaynshet G (2007)(35) | Y | Y | Y | Y | Y | Y | Y | Y | Y | 9 |
| Workye et al.(2023)(40) | Y | Y | Y | Y | Y | Y | Y | Y | Y | 9 |
| Benti and Teferi (2015)(41) | Y | Y | NA | Y | Y | Y | NA | Y | Y | 7 |
| Bekele and Deressa (2014)(25) | Y | Y | Y | Y | Y | Y | Y | Y | Y | 9 |
| Shimekaw et al.(2013)(42) | Y | NA | Y | Y | Y | Y | NA | Y | Y | 7 |
| Temesgan W.Z. et al.(2021)(43) | Y | Y | NA | Y | Y | Y | NA | Y | Y | 7 |
| Mamaru A et al.(2015)(44) | Y | Y | Y | Y | Y | Y | Y | Y | Y | 9 |
| Tegegne KT et al. (2019)(45) | Y | Y | Y | NA | Y | Y | Y | Y | Y | 8 |
| Esayas et al.(2023)(46) | Y | Y | Y | Y | Y | Y | Y | Y | Y | 9 |
| Seid A (2018)(47) | Y | Y | NA | Y | NA | Y | Y | Y | Y | 7 |
| A Takele and T Setegn (2014)(39) | Y | Y | Y | Y | Y | Y | Y | Y | Y | 9 |
| SM Hassen and BH Mohammed (2021)(48) | Y | Y | NA | NA | Y | Y | Y | NA | Y | 6 |
| Tora A (2013)(49) | Y | Y | Y | Y | Y | Y | Y | Y | Y | 9 |
| Kassa S et al.(2019)(50) | Y | Y | NA | Y | Y | Y | NA | Y | Y | 7 |
| Bekele et al.(2015)(51) | Y | Y | Y | Y | Y | Y | Y | Y | Y | 9 |
| Yemsrach et al.(2017)(52) | Y | NA | Y | NA | Y | Y | NA | Y | Y | 6 |
| EG Sendo and M Meleku (2015)(53) | Y | NA | Y | Y | Y | Y | NA | Y | Y | 7 |
| Henock et al.(2015)(54) | Y | Y | Y | Y | Y | Y | Y | Y | Y | 9 |
| Adinew and Hagos (2017)(27) | Y | Y | Y | NA | Y | Y | Y | Y | Y | 8 |
| Tadesse S (2004)(55) | Y | Y | Y | Y | Y | Y | Y | Y | Y | 9 |
| **Key:** **Y**= Yes; **NR**= Not reported, **NA**=Not appropriate | | | | | | | | | | |

Question codes:

1. Was the sample frame appropriate to address the target population?

2. Were study participants sampled in an appropriate way?

3. Was the sample size adequate?

4. Were the study subjects and the setting described in detail?

5. Was the data analysis conducted with sufficient coverage of the identified sample?

6. Were valid methods used for the identification of the condition?

7. Was the condition measured in a standard, reliable way for all participants?

8. Was there appropriate statistical analysis?

9. Was the response rate adequate, and if not, was the low response rate managed appropriately?
